# Evaluation of Whole-Genome Sequence Method to Diagnose Resistance of 13 Anti-tuberculosis Drugs and Characterize Resistance Genes in Clinical Multi-Drug Resistance *Mycobacterium tuberculosis* Isolates From China

**DOI:** 10.3389/fmicb.2019.01741

**Published:** 2019-07-31

**Authors:** Xinchang Chen, Guiqing He, Shiyong Wang, Siran Lin, Jiazhen Chen, Wenhong Zhang

**Affiliations:** ^1^Department of Infectious Diseases, Huashan Hospital, Fudan University, Shanghai, China; ^2^Department of Infectious Diseases, Wenzhou Sixth People's Hospital, Wenzhou Central Hospital Medical Group, Wenzhou, China

**Keywords:** drug resistance, drug susceptibility test, whole-genome sequencing, multidrug-resistant tuberculosis, mutation

## Abstract

**Background:** Whole-genome sequencing (WGS) is a viable and financially feasible tool for timely and comprehensive diagnosis of drug resistance in developed countries. With the increase in the incidence of multidrug-resistant tuberculosis (MDR-TB), second-line anti-TB drugs are gaining importance. However, genetic resistance to second-line anti-TB drugs based on WGS has not been fully studied.

**Methods:** We randomly selected 100 MDR-TB and 10 non-MDR-TB isolates from a hospital in Zhejiang Province, China. Drug susceptibility tests against 13 anti-TB drugs were performed, and 34 drug resistance-related genes were analyzed using WGS in all isolates. For each drug, the accuracy, sensitivity, specificity, and positive and negative predictive values of WGS were compared with those of the conventional drug susceptibility test.

**Results:** The overall sensitivity and specificity for WGS were respectively, 99.0 and 100.0% for isoniazid (INH), 99.0 and 100.0% for rifampicin (RIF), 94.8 and 65.3% for ethambutol (EMB), 86.2 and 84.4% for pyrazinamide (PZA), 95.6 and 95.6% for levofloxacin (LFX), 89.5 and 65.3% for moxifloxacin (MFX), 91.3 and 95.1% for streptomycin (SM), 90.9 and 99.0% for kanamycin, 90.9 and 100.0% for amikacin, 88.9 and 98.0% for capreomycin, 87.0 and 85.1% for prothionamide (PTO), 85.7 and 99.0% for para-aminosalicylic acid (PAS), and 66.7 and 95.9% for clofazimine (CLO).

**Conclusions:** WGS is a promising approach to predict resistance to INH, RIF, PZA, LFX, SM, second-line injectable drugs (SLIDs), and PTO with satisfactory accuracy, sensitivity, and specificity of over 85.0%. The specificity of WGS in diagnosing resistance to EMB, and high-level resistance to MFX (2.0 mg/L) needs to be improved.

## Introduction

In 2017, there were ~558,000 new cases of rifampicin-resistant tuberculosis (RR-TB) worldwide, including 82% multidrug-resistant (MDR-TB) cases with resistance to isoniazid (INH), and rifampicin (RIF). Only 28% of MDR/RR-TB cases have been detected worldwide (World Health Organization, 2018a). GeneXpert MTB/RIF as a sensitive and quick diagnostic tool is the recommended initial diagnostic test to detect pulmonary TB and RIF resistance (World Health Organization, 2013; Denkinger et al., 2014; Steingart et al., 2014). Line probe assays (LPAs) enable rapid drug-susceptibility testing for RIF and INH resistance and *Mycobacterium tuberculosis* detection. However, conventional culture and drug susceptibility tests (DSTs) for second-line agents are currently still indispensable in countries with documented or suspected cases of extensive drug resistant TB (XDR-TB) to ensure quality testing of second-line agents, following the World Health Organization (WHO) policy guidance (Denkinger et al., 2014; Steingart et al., 2014). The cumbersome technology, high infrastructure requirements, and long cycle has made accessibility to DST is low. In China, only 18% cases of DR-TB were documented (World Health Organization, 2018a).

Studies have confirmed the viability and financial feasibility of whole-genome sequencing (WGS) in the diagnosis of clinical resistance to first-line anti-TB drugs, especially in developed countries (Walker et al., 2015; Farhat et al., 2016; Pankhurst et al., 2016; Miotto et al., 2017; Allix-Béguec et al., 2018; Zignol et al., 2018). With the rise in the incidence of DR-TB in China, South Africa, Russia, and other developing countries, second-line anti-TB drugs are becoming increasingly important. WGS can quickly and comprehensively detect all possible drug resistance genes simultaneously, which could provide more information for clinical treatment, especially MDR-TB. However, there are only a few studies on the accuracy of WGS in predicting resistance to second-line anti-TB agents. As the country with the second highest burden of DR-TB worldwide, it would be more meaningful and useful if WGS could accurately diagnose drug resistance to second- and first-line drugs.

Therefore, in this study, we evaluated the performance of a WGS approach for diagnosing drug susceptibility and resistance to 100 MDR-TB, and 10 non-MDR-TB clinical isolates in China. The results of the WGS were compared with phenotypic DST results of 13 anti-TB drugs including drugs less studied but receiving increasing attention [para-aminosalicylic acid (PAS), prothionamide (PTO), and clofazimine (CLO)].

## Materials and Methods

### Clinical Isolates

In total, 100 clinical MDR *Mycobacterium tuberculosis* (MTB) isolates were randomly selected from preserved MDR-TB strains between January 1, 2014 and June 30, 2017 at the Sixth People's Hospital of Wenzhou city, Zhejiang Province, China. To better evaluate test specificities, 10 clinical non-MDR-TB isolates were also selected randomly. This was a retrospective study and the strains included were all stocked strains. All isolates and the reference strain MTB H37Rv were thawed and cultured on Löwenstein–Jensen medium. A colloidal gold assay (Genesis Biodetection and Biocontrol Ltd., Hangzhou, Zhejiang Province, China) to detect MPB64 antigen was used routinely to differentiate MTB from NTM. All NTM isolates were excluded from the study. After extracting the DNA, we confirmed TB further using polymerase chain reaction (PCR) amplification, and Sanger sequencing of 16s rRNA. If a patient had multiple isolates, the earlier isolates were excluded.

### Drug Susceptibility Test

The drug susceptibility tests of all clinical isolates and the reference strain MTB H37Rv to 13 anti-TB drugs were carried out according to the Clinical and Laboratory Standards Institute (CLSI) and World Health Organization (WHO) guidelines. All antibiotics, except pyrazinamide (PZA) and PTO, were tested using the proportion method on commercial Löwenstein–Jensen medium with antibiotics (Baso, Zhuhai, Guangzhou Province, China). The critical concentrations were 0.2 mg/L for INH, 40.0 mg/L for RIF, 2.0 mg/L for ethambutol (EMB), 2.0 mg/L for levofloxacin (LFX), 2.0 mg/L for moxifloxacin (MFX), 4.0 mg/L for streptomycin (SM), 30.0 mg/L for amikacin (AM), 30.0 mg/L for kanamycin (KM), 40.0 mg/L for capreomycin (CM), 1.0 mg/L for PAS, and 1.0 mg/L for CLO, respectively, according to the CLSI guidelines, and WHO guidelines (Institute., CALS, 2011; World Health Organization, 2018b). The results were determined after 3 weeks incubation at 37°C. The susceptibility of MTB to PZA and PTO was evaluated using an automated Mycobacterial Growth Indicator Tube 960 system (Becton Dickinson Diagnostic Systems, Franklin Lakes, NJ, USA) according to the manufacturer's instructions at critical concentrations of 100.0 and 2.5 mg/L, respectively. The well-known limitation of technical feasibility and reproducibility of the phenotypic DST of PZA, EMB, and PTO, required the DST to be performed at least twice for these three drugs. If the two results were inconsistent, a third test was performed. All experiments using live MTB were performed in a biosafety level 2 plus laboratory.

### WGS and Phylogenetic Tree Construction

MTB colonies were scraped from the Löwenstein–Jensen medium and genomic DNA was extracted using the Dneasy Blood & Tissue Kit (QIAGEN, Valencia, CA, USA), following the manufacturer's protocol. All sequencing libraries were prepared using the Nextera XT Sample Prep Kit (Illumina, San Diego, CA, USA) following the manufacturer's protocol and sequenced on Illumina Miseq, Illumina Hiseq, and BGISEQ-500 platforms, with depths of at least 50-fold coverage. After filtering the low-quality reads, the reads were aligned to the MTB H37Rv (GenBank NC000962.3) reference sequence using Bowtie2 (version 2.3.3.1) with default parameters. Single nucleotide polymorphisms (SNPs) were detected using SAMtools (version 1.6) with a minimum sequencing depth of 10 reads without strand bias and a frequency of no <10%.

Since duplicative clones or closely clustered strains would decrease mutation variety and effective statistical sample numbers, considering all 100 MDR strains were collected in one hospital, we built a phylogenetic tree using reliable SNPs of the whole genome to exclude possible genetic simplicity of the isolates. The phylogenetic tree was constructed using the maximum likelihood method using MEGA (version 7.0) software based on the reliable SNPs, with H37Rv as the root. If the mutation ratio of a certain site was above 80%, it was considered a fixed mutation. SNPs in highly repetitive regions such as PE and PPE, GC-enriched sequences, and drug resistance-related genes were removed from the analysis. The strains with a genetic distance of no more than 12 SNPs were considered recent transmission (Walker et al., 2013; Yang et al., 2017).

### Interpretation of Mutations Related to Resistance

The genetic resistance diagnosis of INH (*katG, inhA*, and *ahpC-promoter*), RIF (*rpoB*), PZA (*pncA*), EMB (*embB* and *embA*), LFX (*gyrA* and *gyrB*), MFX (*gyrA*), AM (*rrs*), KM (*eis* and *rrs*), CM (*tlyA* and *rrs*), SM (*rrs* and *rpsL*), PTO (*inhA* and *ethA*), PAS (*folC, thyA, dfrA*, and *ribD*), and CLO (*rv0678*) were based on resistance mutation sites reported in previous studies (Table S1) (Chakraborty et al., 2013; Walker et al., 2015; Zhang et al., 2015a,b; Farhat et al., 2016; Miotto et al., 2017; Allix-Béguec et al., 2018; Liu et al., 2018). Isolates containing resistance mutations were diagnosed as genetically resistant.

In order to find new possible resistance-related mutations, we further analyzed all mutations in genes above and another 15 resistance-related genes for INH (*nat, ndh, iniA, iniB*, and *iniC*), PZA (*rpsA* and *panD*), EMB (*embC, embR*, and *ubiA*), SM (*gidB*), and CLO resistance (*rv1979c, rv2535c, mmpL3*, and *mmpL5*) (Table S1) (Wong et al., 2011; Zhang et al., 2013, 2015a; Hartkoorn et al., 2014; Shi et al., 2014; Vilchèze and Jacobs, 2014; Almeida et al., 2016; Li et al., 2017; Ismail et al., 2018; Sun et al., 2018; Khosravi et al., 2019; Tulyaprawat et al., 2019).

### Statistical Analyses

The phenotypic DST was used as the gold standard to calculate the accuracy, sensitivity, specificity, positive predictive value (PPV), and negative predictive value (NPV), and their 95% confidence intervals of WGS by Stata 13.1. The sensitivity and specificity are the percentages of true positives and negatives, respectively. The sensitivity and specificity are also the proportion of strains that were genetically resistant and sensitive among all phenotypic resistant and sensitive strains, respectively. PPV and NPV were calculated using the relevant formula.

## Results

### Basic and Clustering Information of Strains

In total, 110 strains were reconfirmed as MTB by aligning the sequencing reads to the reference genome of MTB H37Rv. After constructing the phylogenetic tree of the MDR strains, five pairs of isolates were found to be closely related, suggesting recent transmission events (Figure S1). The clustering rate of MDR isolates was 5.0%. The low clustering rate of the isolates excluded the possibility of low genetic variety of the isolates, albeit they were collected in one hospital and, therefore, indicated low risks of over-estimating the weight of specific rare mutations carried by the strains. Among them, 79 MDR and 8 non-MDR-TB strains belonged to lineage 2, and 21 MDR and 2 non-MDR TB strains belonged to lineage 4 (Coll et al., 2014).

### DST

The susceptibility test for all drugs, except CLO, was performed on all clinical isolates; CLO susceptibility test was performed using only 80 isolates because of the low resistance rate. The antibiotic phenotypic resistance rate of all the clinical strains was as follows: INH, 91.8%; RIF, 90.9%; SM, 62.7%; LFX, 59.1%; PZA, 59.1%; EMB, 52.7%; MFX, 34.5%; PTO, 20.9%; AM, 10.0%; KM, 10.0%; CM, 8.2%; PAS, 6.4%; and CLO, 5.0%.

### Comparison of WGS and Phenotypic DST Results

#### Drugs With High Accuracy

##### Inh

Among the 110 strains, 101, and 9 strains were INH-resistant and INH-sensitive, respectively. The most common INH-resistant mutations were KatG S315N/T, *inhA* c-15t, and KatG S315T+*inhA* c-15t, which accounted for 66.3, 11.9, and 5.0% of the phenotypically resistant strains, respectively (Table S2). Four INH-resistant strains had early termination or frameshift mutations in the *katG* gene (Q88^*^, W198^*^, 334 g-in + 713 gcg-in, and 2044 t-in). Six resistant strains carried resistance-associated mutations in the promoter of *ahpC*. According to previous criteria, the accuracy, sensitivity, specificity, PPV, and NPV, and their 95% confidence intervals of the WGS diagnosis for INH were 93.6%, 93.1% (85.6–96.9%), 100.0% (62.9–100.0%), 100.0% (95.1–100.0%), and 56.3% (30.6–79.2%), respectively (Table 1). Seven phenotypically resistant but genetically sensitive strains showed inconsistent results between the DST and WGS according to previous diagnosis criteria. Six of these seven strains carried mutations in *katG*, a well-defined INH resistance-related gene, namely Q88P+M257V, W91R, A122D, Q127P, A312E, and D419Y. After including the new mutations in the diagnosis criteria, the accuracy, and sensitivity improved to 99.1 and 99.0% (93.8–99.9%), respectively. No mutations in *iniB* and *iniC* were detected. Mutations in Ndh (Q52R, A154V, and M138V) and IniA (R479P and D198G) found in six INH-resistant isolates were all present with KatG S315T, except in one phenotypically resistant isolate with only IniA K526R.

**Table 1:** Whole-genome sequencing (WGS) compared with phenotypic drug sensitivity test (DST) in drug resistance diagnosis of *Mycobacterium tuberculosis* (MT).

**Drug**		**Locus**	**Phenotypically resistant**		**Phenotypically sensitive**		**Consistency (%)**	**Sensitivity (%)**	**Specificity (%)**	**PPV (%)**	**NPV (%)**
			**Genetically resistant**	**Genetically sensitive**	**Genetically resistant**	**Genetically sensitive**					
INH		*katG,* *inhA-p,* *aphC-p*	94	7	0	9	93.6	93.1 (85.6–96.9)	100.0 (62.9–100.0)	100.0 (95.1–100.0)	56.3 (30.6–79.2)
INH-improved		*katG,* *inhA,* *aphC-p*	100	1	0	9	99.1	99.0 (93.8–99.9)	100.0 (62.9–100)	100.0 (95.4–100)	90.0 (54.1–99.5)
RIF		*rpoB*	99	1	0	10	99.1	99.0 (93.8–99.9)	100.0 (65.5–100)	100.0 (95.3–100)	90.9 (57.1–99.5)
EMB		*embB,* *embA,* *embC,* *embR,* *ubiA*	55	3	17	35	80.9	94.8 (84.7–98.6)	65.3 (50.8–77.7)	75.3 (63.6–84.4)	91.9 (77.0–97.9)
PZA		*pncA,* *rpsA,* *panD*	56	9	7	38	85.5	86.2 (74.8–93.1)	84.4 (69.9–93.0)	88.9 (77.8–95.0)	80.9 (66.3–90.4)
FQL	LFX	*gyrA,* *gyrB*	59	6	2	43	92.7	95.6 (80.3–96.2)	95.6 (83.6–99.2)	96.7 (87.5–99.4)	87.8 (74.5–94.9)
	MFX	*gyrA*	34	4	23	49	73.6	89.5 (74.3–96.6)	65.3 (53.1–75.9)	57.6 (44.1–70.2)	98.2 (80.3–97.5)
SLID	KM	*rrs,* *eis-p*	10	1	1	98	98.2	90.9 (57.1–99.5)	99.0 (93.7–99.9)	90.9 (57.1–99.5)	99.0 (93.7–99.9)
	AM	*rrs*	10	1	0	99	99.1	90.9 (57.1–99.5)	100.0 (95.3–100.0)	100 (65.5–100.0)	99.0 (93.8–99.9)
	CM	*rrs,* *tlyA*	8	1	2	99	97.3	88.9 (50.7–99.4)	98.0 (92.3–99.7)	80.0 (44.2–96.5)	99.0 (93.8–99.9)
SM		*rpsL,* *rrs*	59	10	2	39	89.1	85.5 (74.5–92.5)	95.1 (82.2–99.2)	96.7 (87.6–99.4)	79.6 (65.2–89.3)
SM-improved		*rpsL,* *rrs,* *gidB*	63	6	2	39	92.7	91.3 (81.4–96.4)	95.1 (82.2–99.2)	96.9 (88.4–99.5)	86.7 (72.5–94.5)
PTO		*ethA,* *inhA-p*	20	3	13	74	85.5	87.0 (65.3–96.6)	85.1 (75.4–91.5)	60.6 (42.2–76.6)	96.1 (88.3–99.0)
PAS		*folC,* *thyA,* *ribD,* *dfrA*	6	1	1	102	98.2	85.7 (42.0–99.2)	99.0 (93.9–99.9)	85.7 (42.0–99.2)	99.0 (93.9–99.9)
CLO		*rv0678,* *rv1979c,* *rv2535c,* *mmpL3 and mmpL5*	4	2	3	71	93.8	66.7 (24.1–94.0)	95.9 (87.8–98.9)	57.1 (20.2–88.2)	97.3 (89.6–99.5)

*INH, isoniazid; RIF, isoniazid; EMB, ethambutol; PZA, pyrazinamide; LFX, levofloxacin; MFX, moxifloxacin; SM, streptomycin; AM, amikacin; KM, kanamycin; CM, capreomycin; PTO, prothionamide; PAS, para-aminosalicylic acid; CLO, clofazimine; –p, promoter*.

##### Rif

Genetic resistance against RIF was mostly diagnosed as mutations in the 81-bp *rpoB* RIF resistance-determining region (RRDR). The most prevalent drug-resistant mutations were S450L/F/W, D435V, H445D/L/Y, L452P, and Q432L, which accounted for 64.0, 13.0, 7.0, 1.0, and 2.0%, respectively, among 100 phenotypically resistant strains. There were also six strains with double mutations, namely V170F+H445Y, L430P+D435G, L430P+D435N, L430P+H445N, D435A+H445D, D435A+L452P, and H445Q+L452S. One isolate carried *rpoB* H674Y+1291 gcc-in causing frameshift in RRDR and was considered RIF-resistance. Only one isolate with inconsistent results between the WGS diagnosis and phenotypic DST had no mutation in the *rpoB* gene (Table S3). The accuracy, sensitivity, specificity, PPV, and NPV of RIF WGS diagnosis were 999.1%, 99.0% (93.8–99.9%), 100.0% (65.5–100.0%), 100.0% (95.3–100.0%), and 90.9% (57.1–99.5%), respectively (Table 1).

##### pza

PZA resistance is associated with full-length mutations in *pncA* (Yadon et al., 2017; Liu et al., 2018). Among the 65 resistant strains, 86.2% (56/65) strains carried mutation in *pncA* (Table S5). We also screened mutations in *rpsA* and *panD*, which are associated with resistance to PZA (Zhang et al., 2013; Shi et al., 2014). Mutations in *rpsA* were found in seven resistant strains, which all had a *pncA* mutation; no mutation was detected in *panD*. We included all mutations that cause amino acid changes in pyrazinamidase (encoded by *pncA*) as the resistance diagnosis criteria, and the accuracy, sensitivity, specificity, PPV, and NPV of the WGS diagnosis were 85.5%, 86.2% (74.8–93.1%), 84.4% (69.9–93.0%), 88.9% (77.8–95.0%), and 80.9% (66.3–90.4%) (Table 1).

Six new mutations in *pncA* were identified including PncA A28D, D136G, M175R, T177P, and S59Y+T153P. The same position but different amino acid mutations, D136Y and M175I/V, were previously detected in PZA-R strains, which further supported the notion that these new mutations result in PZA resistance. Totally, nine phenotypically resistant but genetically sensitive isolates had no mutations in *pncA, panD*, and *rpsA*, indicating undiscovered mechanisms of resistance. For seven phenotypically sensitive but genetically resistant strains, four strains carried PncA L35R, T47I, T47P, and F106C respectively, among which L35R, T47P, and F106C have not been found in PZA-R isolates before.

##### lfx

For LFX, the most common drug-resistant mutations occurred in the quinolone resistance determining region (QRDR) of *gyrA* (Mayer and Takiff, 2014). The most frequent drug-resistant mutations were D94A/G/H/N/Y, A90V, and S91P, which accounted for 49.2, 24.6, and 4.6%, respectively. Additionally, nine (13.8%) resistant isolates, namely G88A+D94A, A90V+D94A, A90V+D94H, A90V+D94G, A90V+D94Y, two each with A90V+D94N and S91P+D94A, contained two common drug-resistant mutations in QRDR. Among the other six phenotypically resistant strains, three were genetic wildtype and the other three strains carried mutations of QRDR, namely GyrA G158V+GyrB D461N, GyrB V636L, and GyrA I287T. Two phenotypically sensitive strains carried GyrA A90V and D94A mutations (Table S6).

The resistance-associated region in GyrB was codons 500–540. Nine strains carrying the T500A, E501V, E501D, A504V, and G512R mutations were found, but all occurred simultaneously with GyrA A90V, or D94G/N. The accuracy, sensitivity, specificity, PPV, and NPV of LFX WGS diagnosis were 92.7%, 95.6% (80.3–96.2%), 95.6% (83.6–99.2%), 96.7% (87.6–99.4%), and 87.8% (74.5–94.9%), respectively (Table 1).

##### Second-line-injectable-drugs-slid

A total of 10 strains carried *rrs*-a1401g, the most prevalent resistance-related mutations of second-line injectable drugs (SLIDs). All 10 strains with *rrs*-a1401g were phenotypically resistant to AM and KM, whereas 8 out of the 10 were phenotypically resistant to CM. One strain with a resistant phenotype did not have any known resistance-related mutation (Tables S8–S10). One isolate with *eis* (c-10t), which was reported to cause KM resistance, was phenotypically susceptible to KM. For AM, the accuracy, sensitivity, specificity, PPV, and NPV of the WGS diagnosis were 99.1%, 90.9% (57.1–99.5%), 100.0% (95.3–100.0%), 100.0% (65.5–100.0%), and 99.0% (93.8–99.9%), respectively. For KM, the accuracy, sensitivity, specificity, PPV, and NPV of WGS diagnosis were 98.2%, 90.9% (57.1–99.5%), 99.0% (93.7–99.9%), 90.9% (57.1–99.5%), and 99.0% (93.7–99.9%), respectively. For CM, the accuracy, sensitivity, specificity, PPV, and NPV of WGS diagnosis were 97.3%, 88.9% (50.7–99.4%), 98.0% (92.3–99.7%), 80.0% (44.2–96.5%), and 99.0% (93.8–99.9%), respectively (Table 1).

Among the 69 SM-resistant strains, the most common drug-resistant mutations were RpsL K43R, K88R, *rrs* a514c, and RpsL K43T+*rrs* a514c, which accounted for 71.0, 7.2, 5.8, and 1.4%, respectively. Two other strains carrying RpsL K43R or *rrs* a514c mutation were phenotypically sensitive to SM (Table S11). Mutations in the *gidB* gene were diverse and were reported to result in low-level resistance to SM (Tudó et al., 2010; Wong et al., 2011). Except for frameshift mutations, few SNP mutations have been identified to cause SM resistance. We found five new frameshift mutations of *gidB* including 102g_del, 351g_del, 386g_in, 115c_del, and 114c_del (together with RpsL K43R) in 5 SM-R isolates, respectively. After including frameshift mutations of *gidB* in the diagnosis criteria, the accuracy and sensitivity of the SM WGS diagnosis increased from 89.1 and 85.5% (74.5–92.5%) to 92.7 and 91.3% (81.4–96.4%), respectively (Table 1).

##### Pto

PTO resistance is mainly associated with mutations in *ethA* and the promoter of *inhA* (Morlock et al., 2003; Brossier et al., 2011; Vilchèze and Jacobs, 2014). In this study, 23 and 87 strains were PTO phenotype resistant and sensitive, respectively. The mutation of *inhA* (c-15t) alone without mutation in *ethA* was found in 13.0% PTO-resistant and 4.6% PTO-susceptible strains. Double mutation of the *inhA* (c-15t) and *ethA* genes (*ethA* t-11c, 1114t-del, EthA W45R, R54H, W116C, G184D, and L190P) was detected in 34.8% of the PTO-resistant and 1.1% PTO-susceptible strains (EthA T342A, Table S12). The previous drug resistance diagnostic criteria only included *inhA* (Miotto et al., 2017). EthA is the activator of PTO; thus, from the perspective of mechanism, mutations in *ethA* that result in loss of EthA function could lead to drug resistance (Vilchèze and Jacobs, 2014). Therefore, pooled frameshifts and premature stop codons were included in our diagnostic criteria (Table S1). The accuracy, sensitivity, specificity, PPV, and NPV of the PTO WGS diagnosis were 85.5%, 87.0% (65.3–96.6%), 85.1% (75.4–91.5%), 60.6% (42.2–76.6%), and 96.1% (88.3–99.0%), respectively (Table 1).

#### Drugs With Moderate Accuracy

##### Emb

There were 58 and 52 strains with EMB phenotype resistance and sensitivity, respectively. The most common drug-resistant mutations were EmbB M306V and M306I, which were found in 21 and 16 of 58 resistant isolates, respectively. However, these two mutations were also detected in 3 and 5 of 52 sensitive isolates. Table 2 and Table S4 show the distribution of these mutations in EMB-resistant and -susceptible strains. The accuracy, sensitivity, specificity, PPV, and NPV of EMB WGS diagnosis were 80.9%, 94.8% (84.7–98.7%), 65.3% (50.8–77.7%), 75.3% (63.6–84.4%), and 91.9% (77.0–97.9%), respectively (Table 1). Most mutations in *embA, embC, embR*, and *ubiA* found in EMB resistance isolates were present together with mutations in *embB* (Table S4). Only two out of three strains with phenotypic resistance to EMB but genetic sensitivity carried EmbA V206M+A542V, and *embC* g-43c mutations, respectively. The other phenotypically resistant isolate carried EmbB M306L.

**Table 2:** Distribution of mutations in *embB* and *embA* in 58 EMB-resistant and 52 susceptible strains.

***embB***	***embA***	**Resistant isolates with mutations = 58**	**Resistant isolates with mutations (%)**	**Sensitive isolates with mutations = 52**	**Sensitive isolates with mutations (%)**
	**c-8t**	0	0.0	1	1.9
	**c-16t**	0	0.0	3	5.8
**D328Y**		2	3.4	0	0.0
**D354A**		4	6.8	1	1.9
**M306I**		14	24.1	4	7.7
M306L		1	1.7	1	1.9
M306L	**c-16t**	1	1.7	0	0.0
**M306V**		21	36.2	3	5.8
**G406A**		1	1.7	1	1.9
**G406C**		2	3.4	0	0.0
**G406D**		2	3.4	1	1.9
**G406S**		1	1.7	1	1.9
**Q497K**		2	3.4	1	1.9
**Q497R**		1	1.7	1	1.9
**Q497P**		1	1.7	0	0.0
**M306I+G406A**		1	1.7	0	0.0
**M306I+G406D**		1	1.7	0	0.0
**M306I+Q497R**		0	0.0	1	1.9
M306L+**G406D**		1	1.7	0	0.0
WT	WT	0	1.7	21	40.4
Other mutations#		2	5.2	12	23.1

*The mutations indicated in bold are related to phenotypic resistance. #A detailed list can be found in Table S5 (EMB, ethambutol)*.

##### Mfx

Among the 65 strains resistant to LFX, 38 were also resistant to MFX. For MFX, the most common resistance mutation sites were GyrA A90V, D94G, D94H, D94N, and D94Y. The distribution of the above sites in resistant and susceptible strains is shown in Table 3. Outside the QRDR, the phenotype of the one isolate carrying GyrA I287T was resistant (Table S7). The accuracy, sensitivity, specificity, PPV, and NPV of the WGS diagnosis were 73.6%, 89.5% (74.3–96.6%), 65.3% (53.1–75.9%), 57.6% (44.1–70.2%), and 98.2% (80.3–97.5%), respectively (Table 1).

**Table 3:** Distribution of mutations in *gyrA* in MFX-resistant and LFX-resistant strains.

	**MFX**		**LFX**	
**Codon**	**resistant isolates (number/ percentage)**	**sensitive isolates (number/ percentage)**	**resistant isolates (number/ percentage)**	**sensitive isolates (number/ percentage)**
**A90V**	4 (10.5%)	12 (16.7%)	15 (23.1%)	1 (2.2%)
S91P	1 (2.6%)	2 (2.8%)	3 (4.6%)	
**D94A**	2 (5.3%)	4 (5.6%)	5 (7.7%)	1 (2.2%)
**D94G**	11 (28.9%)	5 (6.9%)	16 (24.6%)	
**D94N**	8 (21.1%)		8 (12.3%)	
D94H	1 (2.6%)	1 (1.4%)	2 (3.1%)	
**D94Y**	1 (2.6%)	1 (1.4%)	2 (3.1%)	
**A90V+D94A**	1 (2.6%)		1 (1.5%)	
**A90V+D94G**	1 (2.6%)		1 (1.5%)	
**A90V+D94N**	2 (5.3%)		2 (3.1%)	
**A90V**+D94H	1 (2.6%)		1 (1.5%)	
**A90V+D94Y**		1 (1.4%)	1 (1.5%)	
S91P+**D94A**	2 (5.3%)		2 (3.1%)	
Other mutations	1 (2.6%)	7 (9.7%)	3 (4.6%)	4 (8.9%)
WT	2 (5.3%)	39 (54.2%)	3 (4.6%)	39 (86.7%)
	38	72	65	45

*The mutations indicated in bold are related to phenotypic resistance to MFX. LFX, levofloxacin; MFX, moxifloxacin*.

*^#^: A detailed list can be found in Tables S6, S7*.

#### Drugs With Insufficient Evaluation

##### Pas

PAS resistance is mainly related to the folate metabolism pathway, and the main genes involved include *thyA, folC, ribD*, and *dfrA* (Mathys et al., 2009; Chakraborty et al., 2013; Zhao et al., 2014; Zhang et al., 2015b; Cheng et al., 2016). In this study, the seven resistant strains carried the following mutations: two strains with FolC I43T, one strain with FolC I43T+*thyA* t-31c, two strains with FolC S150G, one strain with FolC S150G+ThyA H75N, and one wildtype strain. Of the 103 sensitive strains, 10 carried mutations in the above-mentioned genes, namely DfrA Q28L, DfrA E90A, FolC F374L (three strains), FolC G283D, *thyA* t-31c, ThyA S160G, *thyA* 290g_in, and FolC S150G+ThyA A262V (Table S13). Resistance-related mutations reported previously were included in the diagnostic criteria (Table S1), and the accuracy, sensitivity, and specificity of the WGS diagnosis were 98.2%, 85.7% (42.0–99.2%), and 99.0% (93.9–99.9%), respectively.

##### Clo

Resistance to CLO is mainly related to *rv0678, rv1979c, rv2535c, mmpL3*, and *mmpL5*, of which *rv0678* is the most reported gene in which mutations occur (Hartkoorn et al., 2014; Zhang et al., 2015a; Almeida et al., 2016; Li et al., 2017). The distribution of *rv0678* mutations in drug-resistant strains was as follows: among six phenotypically resistant isolates, two carried L32S, one each carried R50Q and Q115P mutations, and the other two isolates were genetic wildtypes. There were 74 phenotypically sensitive isolates, consisting of 71 wild type, and 1 each carrying R50Q, E104G, and V1L (Table S14). According to a previous study, CLO resistance-related mutations are dispersed throughout the *rv0678* gene (Zhang et al., 2015a). Therefore, we suggest that as long as a mutation occurs in *rv0678*, it could be judged as resistant. The accuracy, sensitivity, and specificity of the WGS diagnosis were 93.8%, 66.7% (24.1–94.0%), and 95.9% (87.8–98.9%), respectively. However, the positive predictive value was only 57.1% (20.1–88.2%) due to the low resistance rate and unclear mechanism of resistance. After analysis of *rv1979c, rv2535c, mmpL3*, and *mmpL5*, we found Rv1979c T265A in 1 CLO-resistant strain, which occurred simultaneously with Rv0678 Q115P and 10 mutations in 13 CLO-sensitive strains, namely MmpL3 W731G, Q435E, and D152H, MmpL5 Q666H, L367P, G246S, and H77Y, Rv1979c R348Q, and V221G, Rv2535c G306E (Table S14).

### WGS Analysis of Non-MDR

We analyzed 34 resistance-related genes among 10 non-MDR isolates and identified mutations in four isolates, among which one was INH mono-resistant, one was resistant to SM and PZA and the other 8 were pan-susceptible. The INH mono-resistant isolate carried a mutation in *inhA* c-15t, which caused cross-resistance to both PTO and INH. One isolate resistant to SM and PZA carried only one mutation in RpsL K43R. The other two isolates were phenotypically sensitive to all drugs, but they carried mutations in resistance-related genes, namely *pepQ* c-3t and UbiA P122R, respectively (Table S15).

## Discussion

In this study, we compared the results of phenotypic DST and WGS for 13 anti-TB drugs. Among the 13 drugs, DST profiles of LFX, MFX, AM, KM, CM, SM, PTO, CLO, and PAS have not been extensively studied using the WGS method. The data showed that the accuracy, sensitivity, and specificity of WGS in diagnosing the resistance against INH, RIF, PZA, LFX, AM, KM, CM, SM, and PTO were more than 85.0%, suggesting that WGS is a promising tool to predict resistance to these drugs. The highly consistent results of the WGS and DST for first-line anti-TB drugs, namely INH, RIF, and PZA and common second-line anti-TB drugs, namely LFX, AM, KM, CM, SM, were consistent with previously reported resistance profiles (Walker et al., 2015; Allix-Béguec et al., 2018).

WGS has several advantages over traditional DST (Miotto et al., 2017; Allix-Béguec et al., 2018). Firstly, WGS can quickly and comprehensively detect all possible drug resistance genes simultaneously. Therefore, for MDR-TB patient, DST results can be obtained for more drugs to provide an optimal treatment regimen and monitor therapy, or adjust the regimen based on WGS after a side effect. Furthermore, WGS can identify new resistance-related mutations. Secondly, it is easier to deliver strain DNA or inactivated strains to a center laboratory with WGS capabilities than to deliver live TB strains to perform DST, considering the current low accessibility to second-line drugs DST in China and other developing countries because of its difficulty and high infrastructure requirements (World Health Organization, 2018a). Thirdly, it has potential to be time-conserving and economical in the near future, if technically sound. WGS can decrease diagnostic time to 5–15 days in practice compared to the 2–6 weeks for traditional DST. Furthermore, the current commercial sequencing service for MTB WGS is $80–100 per isolate in China, as it is comparable to or slightly more economical than DST for MDR patients who need to undergo all second-line drug tests.

In our study, new or rarely reported mutations in known resistance-related genes were identified. In detail, mutations of INH-resistant isolates containing KatG A122D, and M257V have not been reported and mutations such as Q88P, M257I/T, W91R, Q127P, A312E, and D419Y have been rarely reported (Vilchèze and Jacobs, 2014; Allix-Béguec et al., 2018). One phenotypic resistant but genetic sensitive strain carried IniA K526R. Although *iniA* overexpression may lead to lower susceptibility to INH (Alland et al., 2000; Colangeli et al., 2005), the consequence of mutations in IniA K526R to INH resistance require further validation. Another mutation in Nat G207R was found in both susceptible and resistant strains in previous studies (Upton et al., 2001; Ramaswamy et al., 2003), whereas in our study, it was found in all 14 strains belonging to lineage 4.4, indicating it could be a lineage marker instead of resistance-related mutation.

For EMB, two phenotypically resistant but genetically sensitive isolates carried EmbA V206M+A542V and *embC* g-43c, respectively. Since EmbA V206M was reported as a phylogenetic SNP (Walker et al., 2015; Allix-Béguec et al., 2018), EmbA A542V might be a new EMB resistance-related mutation. E*mbC* g-43c mutation was found previously in both EMB-R and EMB-S isolates (Walker et al., 2015; Allix-Béguec et al., 2018); therefore, the role of this mutation in EMB resistance is complex and requires further study. New PncA mutations including A28D, D136G, M175R, T177P, and S59Y+T153P and frameshift mutations of *gidB* including 102g, 351g, 386g, 115c, and 114c deletion or insertion were identified as PZA or SM resistance-related mutations.

For PTO, since the *inhA* c-15t mutation is well-known to cause PTO resistance as reported in a series of published studies (Morlock et al., 2003; Tan et al., 2017), the previous diagnostic criteria only included *inhA* c-15t (Miotto et al., 2017). EthA activates PTO by acting as a monooxygenase (Wang et al., 2007); therefore, a mechanism involving loss of function of EthA could also result in resistance to PTO (Brossier et al., 2011). After we included pooled frameshifts and premature stop codons of EthA in the drug resistance diagnosis list, the accuracy, sensitivity, and specificity of the WGS reached above 85%. In addition to frameshift and premature stop codon mutations, point mutations with amino acid changes in EthA were found in 24.1% (21/87) of sensitive and 17.4% (4/23) of resistant isolates. Interestingly, we found that a frameshift mutation or early termination of *ethA* did not necessarily lead to phenotypic resistance (Table S11). Furthermore, 30 of the 87 (34.5%) strains with *ethA* gene mutations were still phenotypically sensitive to PTO. This phenomenon may have been due to the presence of multiple monooxygenases in MTB, which likely compensated for the inactivation of EthA (Morlock et al., 2003).

For EMB and MFX (2.0 mg/L), our results suggested that the WGS approach would not adequately replace DST but might have potential after clearer interpretation of gene mutations are achieved in affecting DST. For EMB, the specificity is lower than expected (65.3%, 50.8%−77.7%), as mutations like EmbB M306I, M306V, G406, and Q497 were detected in both sensitive and resistant strains. The phenomenon was also reported in previous studies (Lee et al., 2004; Engström et al., 2012; Cheng et al., 2014). Mutations like M306I only causing a slight increase in MIC, which is sometimes not enough to be diagnosed as resistance in phenotypic DST, might explain the inconsistency (Starks et al., 2009; Plinke et al., 2011). For isolates with contradictory result between WGS and DST, the DST was repeated at least twice to confirm. The unsatisfactory consistency of results for EMB may also be due to inappropriate critical concentrations and poor repeatability of phenotypic DST.

For MFX, the inconsistency between WGS and DST was mainly due to the mutation of GyrA A90V, which was found in 4 (10.5%) resistant and 12 (16.7%) sensitive isolates. GyrA A90V mutation was reported to cause a high level of LFX resistance but low level of MFX resistance (Sirgel et al., 2012; Mayer and Takiff, 2014). Other mutations in QRDR, including D94A, D94G, and D94Y were also found in both MFX-resistant and -sensitive isolates, which lowers the specificity of the diagnosis of MFX resistance.

Because of the low resistance rate and limited number of strains, only six CLO- and seven PAS-resistant strains were included in the study. Therefore, although the sensitivities and specificities were rather high, the result of the WGS diagnosis of CLO and PAS resistance was not convincing. To accurately evaluate the performance of WGS in predicting the DST of CLO and PAS, more resistant clinical strains, especially from patients who used these two drugs, are needed in the future studies. For CLO, apart from *rv0678*, mutations in *rv1979c, rv2535c, mmpL3*, and *mmpL5* were less reported and vague in contribution to CLO resistance. In our study, 11 mutations were detected in CLO-sensitive isolates, which have not been reported. A possible explanation for these phenomena could be that these mutations increased the resistance slightly and were not detected in our study.

The main reasons for the discrepancy between WGS and DST are as follows in our study. Firstly, the mechanism of drug resistance is not clear, and there are still several unknown resistance-related mutations related to PAS and CLO. Secondly, some drugs such as PZA and EMB have poor DST consistency and repeatability. DST of these drugs was performed more than once to mitigate these limitations. For PZA, the PncA V9A, or V139A mutation were detected in both phenotypically resistant and sensitive isolates. According to previous studies, PncA V9A resulted in susceptibility *in vitro* but resistance *in vivo* and PncA V139G/L was found in PZA-R strains (Yadon et al., 2017; Allix-Béguec et al., 2018). Instability of PZA DST and inconsistent results of the *in vivo* and *in vitro* DST may explain the discrepancy. Thirdly, it is recognized that the resistance of MTB to PTO and PZA is related to the *ethA* and *pncA* genes, but the resistance-related mutations are scattered throughout the gene without a hot spot, which makes it difficult to interpret resistance through the mutation site. Finally, heterogeneous resistance has frequently been found in the *gyrA* and *ethA* genes. Resistance-related mutations and wild-type alleles occur simultaneously, making it difficult for phenotypic DST to reflect true susceptibility results, which underscores the value of genetic sequencing over DST (van Rie et al., 2005; Sun et al., 2012).

Our study has some limitations. First, China has a vast territory, and this was a single-center study with a limited number of strains. Thus, the strains selected in this study are not wholly representative of China. Second, because of the low resistance rate, the results are underpowered for some drugs. For example, the resistance rate of AK, KM, PAS, and CLO was <10%, leading to a high false measurement index; therefore, the high specificity of these drugs might not be representative. We have to admit that the study is particularly unconvincing for the drugs with low resistance. On the other hand, for INH, and RIF, the small number of non-resistant strains affected the ranges of the CI and NPV. Thirdly, some mutations, such as EmbB M306I, only cause low-grade drug resistance (Plinke et al., 2011), and the use of a critical concentration method for DST may lead to the misdiagnosis of these resistances, which could also be one of the reasons for the inconsistency between phenotype and genotype in this study. Minimum inhibitory concentration (MIC) test could be better for studying drug resistance methods. However, commercial microplates were not available when the study was carried out. Considering the amount of drugs, numbers of isolates, higher contamination rates, and low quality control of in-house microliter plates, we determined to perform DST mostly using commercial L-J media.

In conclusion, WGS is a promising approach to predict resistance to drugs such as INH, RIF, PZA, LFX, AM, KM, SM, SM, and PTO with a satisfactory accuracy, sensitivity, and specificity of over 85.0%. The specificity of WGS diagnosis for EMB and MFX (2.0 mg/L) was not high enough and needs to be improved. For other drugs such as PAS and CLO, inclusion of more strains, especially resistant strains, is needed to improve data interpretation before widespread clinical adoption.

## Data Availability

The raw data of WGS has already been submitted to the SRA database and can be accessed by the public at https://www.ncbi.nlm.nih.gov/bioproject/PRJNA522942. The BioProject accession number is PRJNA522942. The SRA accession numbers for 110 isolates are from SAMN10961303 to 10961412.

## Author Contributions

All persons who meet authorship criteria are listed as authors, and all authors certify that they have participated sufficiently in the work to take public responsibility for the content, including participation in the concept, design, analysis, writing, or revision of the manuscript. JC and WZ: conception and design of study, revising the manuscript critically for important intellectual content. XC, GH, SW, and SL: acquisition of data, analysis and interpretation of data. XC and JC: drafting the manuscript.

### Conflict of Interest Statement

The authors declare that the research was conducted in the absence of any commercial or financial relationships that could be construed as a potential conflict of interest.
