# Corrigendum: Evaluation of Whole-Genome Sequence Method to Diagnose Resistance of 13 Anti-tuberculosis Drugs and Characterize Resistance Genes in Clinical Multi-Drug Resistance *Mycobacterium tuberculosis* Isolates From China

**DOI:** 10.3389/fmicb.2019.02221

**Published:** 2019-09-24

**Authors:** Xinchang Chen, Guiqing He, Shiyong Wang, Siran Lin, Jiazhen Chen, Wenhong Zhang

**Affiliations:** ^1^Department of Infectious Diseases, Huashan Hospital, Fudan University, Shanghai, China; ^2^Department of Infectious Diseases, Wenzhou Sixth People's Hospital, Wenzhou Central Hospital Medical Group, Wenzhou, China

**Keywords:** drug resistance, drug susceptibility test, whole-genome sequencing, multidrug-resistant tuberculosis, mutation

In the published article, there was an error in affiliation **2**. Instead of “**Sixth People's Hospital of Wenzhou City, Shenzhen, China**,” it should be “**Department of Infectious Diseases, Wenzhou Sixth People's Hospital, Wenzhou Central Hospital Medical Group, Wenzhou, China**.”

The authors apologize for this error and state that this does not change the scientific conclusions of the article in any way. The original article has been updated.

