# Supplementary material for: Evaluation of Whole-Genome Sequence Method to Diagnose Resistance of 13 Anti-tuberculosis Drugs and Characterize Resistance Genes in Clinical Multi-Drug Resistance Mycobacterium tuberculosis Isolates From China
Source: Front Microbiol. 2019 Jul 31;10:1741. doi: 10.3389/fmicb.2019.01741 (PMC6685394; doi:10.3389/fmicb.2019.01741)
Supplement: Supplementary file 1 [file Table_1.DOCX]

***Supplementary Material***

| Table S1 Diagnostic criteria of genetic sequencing for genotypic resistance | | | |
| --- | --- | --- | --- |
| Drugs | Resistance –related genes | Resistance-related mutations | added mutations in this study |
| INH^[1-4]^ | *katG* | W90R, R104Q, A109V, G125D, L141F, D142G, L159P, T180K, G182R, W191G/R, P232R, G297V, W300C, S315I/N/T, W328L, S481L, A614E, V633A, S700P, L704S, pooled frameshifts and premature stop codons | Q88P+M257V, W91R, A122D, Q127P, A312E, D419Y |
|  | *inhA* | t-8c, c-15t, g-17t, I21T, S94A, I194T | / |
|  | *ahpC* | g-48a, c-57t, c-72t | c-52t |
|  | *nat* | Y188H |  |
|  | *ndh* | R13C, T110A, R268H |  |
|  | *iniA* | P3A, R537H |  |
|  | *iniB* | Pooled framshift |  |
|  | *iniC* | t79ins, a98ins, W83G |  |
| RIF^[1-3, 5]^ | *rpoB* | V170F, V359A, L430P, S431T, Q432K/L/P, M434I, D435A/F/G/N/V/Y, S441L/Q, H445C/D/F/G/L/N/P/R/Y, S450F/L/Q/W/Y, L452P, I491F, D545E, T676P, G981D | S450V, S450P, S450H, H445Q+L452S, 1291_gcc_in |
| EMB^[1-3, 5-12]^ | *embB* | M306I/V, D328Y, D354A, G406A/D/S/C, Q497K/R/P, N1033K | / |
|  | *embA* | c-12t, c-16g, c-16t | / |
|  | *embC* | T270I, D329G, N394D | / |
|  | *embR* | P49A+P243S | / |
|  | *ubiA* | L31P, A35E/S, A38V, V55G/M, V148A, G165C, S173A, K174R, W175G, F176L, I179T, M180V, V188A, V229G, L235P, A237V, R240C, S244T, A249G, A278V | / |
| PZA^[2, 3, 13-18]^ | *pncA* | any amino acid change, pooled frameshifts and premature stop codons | / |
|  | *panD* | M117T, E126*, A128S, E130G, P134S, L136R, V138A/G/E, M171I | / |
|  | *rpsA* | / | / |
| FQ^[2]^ |  |  |  |
| LFX | *gyrA* | G88A, G88C, S91P, A90V, D94A, D94G, D94H, D94N, D94Y | / |
|  | *gyrB* | E459K, A504V | / |
| MFX | *gyrA* | G88C, A90V, S91P, D94A, D94G, D94N, D94Y | / |
| SM^[2, 3, 19]^ | *rpsL* | K43R, K43T, K88Q, K88R, T40I | / |
|  | *rrs* | a514c, a514t, c462t, c513t, c517t |  |
|  | *gidB* | / | Pooled framshifts |
| SLI^[2, 3]^ |  |  |  |
| AM | *rrs* | a1401g, g1484t | / |
| KM | *eis* | c-14t, g-10a | / |
|  | *rrs* | a1401g, c1402t, g1484t | / |
| CM | *rrs* | a1401g, c1402t, g1484t | / |
|  | *tlyA* | N236K, pooled frameshifts and premature stop codons | / |
| PTO^[2, 4, 20]^ | *inhA* | c-15t, c-15t+I194T, c-15t+S49A | / |
|  | *ethA* | / | pooled frameshifts and premature stop codons |
| PAS^[21-24]^ | *folC* | / | E40G/K/Q, I43A/F/S/T/V, R49P/W, L56V, N73S, R91W D111A, G112S, D135A, S150G/C/R, F152S/L,E153A/G, V256A, S335I, R410W, A420V, E434Q, A457V |
|  | *thyA* | / | G15R, T22I, Y36C, H75N, G76*, V77F, W83C/*, G91E/R, W98*, S105P, R126Q, F152V, C161T, L183V, R127L, N134K, L143P, L146R, H147N, L172P, A182P, Q191R, H207R, I211V, P224L, R235P, 1A259P, V261G, V263I/G, R264* |
|  | *dfrA* | / | / |
|  | *ribD* | / | g-11a, G8R |
| CLO^[25-29]^ | *rv0678* | / | any amino acid change, pooled frameshifts and premature stop codons |
|  | *rv1979c* | V351A | / |
|  | *rv2535c (pepQ)* | pooled frameshifts and premature stop codons | / |
|  | *mmpL3* | / | / |
|  | *mmpL5* | / | / |

For CLO, the resistance mutations found in drug-resistant strains in previous stydy are as follows: G193-del/in, c364-in, t29-in, a292-del, cg444-445-del, V20F, T33N, A36V, W42*, L43R, C46Y, Q51R, S53L, A59V, S63N, G65E, G66V, S68G, Q76*, A84E, R89L, R90P, A102T/V, L114P, L122P, R156*

Supplemetary figure 1


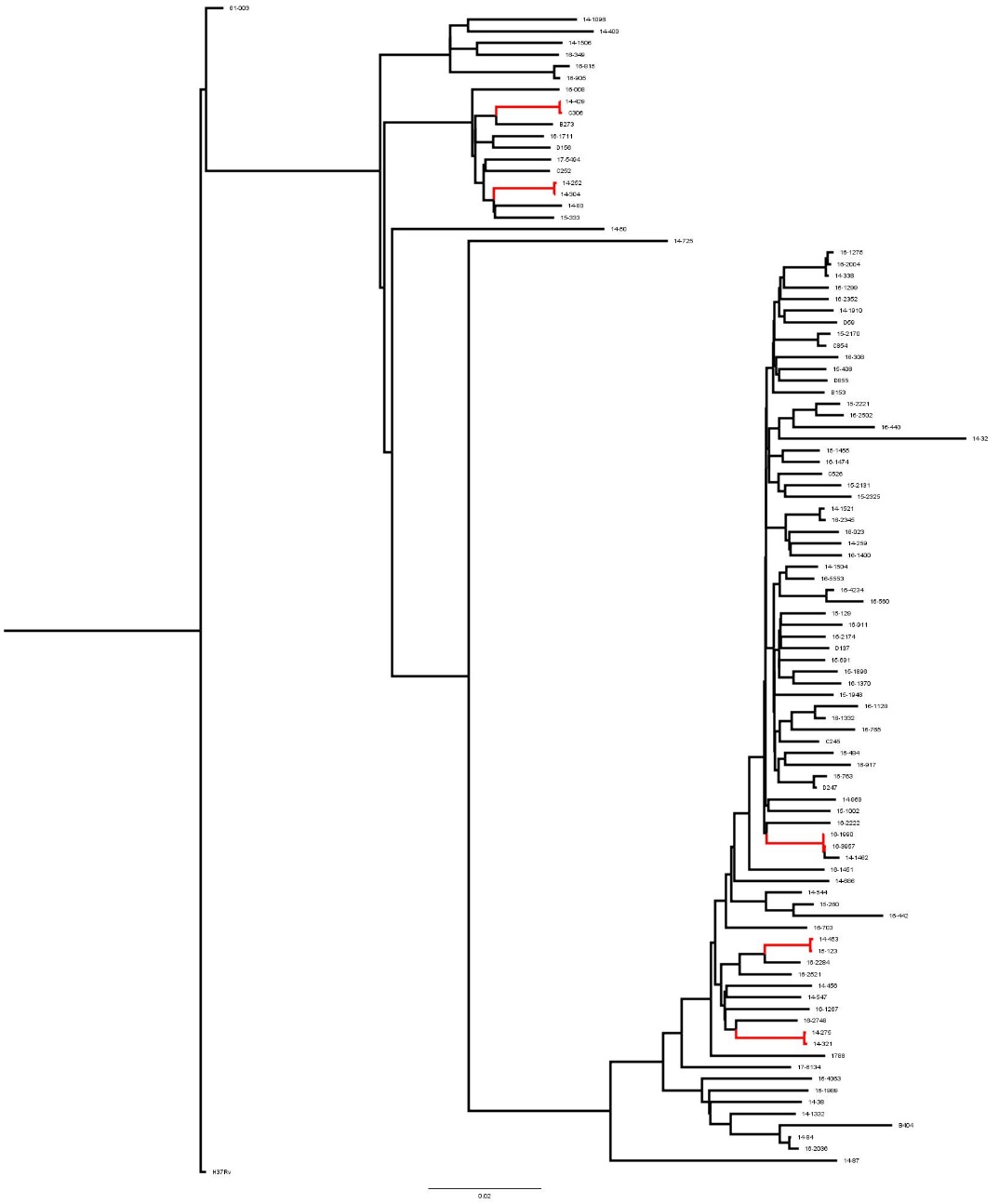


Remark: The isolates with branches highlighted with red are closely related, suggesting transmission events.

Supplement table 2 The distribution of mutations on *katG, inhA, aphC, nat, ndh, iniA, iniB,* and *iniC* in 101 INH-resistant and 9 susceptible strains.

| *inhA*-promoter mutation (codon position) | *katG* mutation (codon position) | *aphC* mutation (codon position) | *ndh* mutation (codon position) | *nat* mutation (codon position) | *iniA* mutation (codon position) | *iniB* mutation (codon position) | *iniC* mutation (codon position) | No. of isolates with resistant phenotype=101 | No. of isolates with susceptible phenotype=9 |
| --- | --- | --- | --- | --- | --- | --- | --- | --- | --- |
| **c-15t** |  | g-51a |  |  |  |  |  | 1 |  |
| **c-15t** |  |  |  |  |  |  |  | 5 |  |
| **c-15t+**A124V |  |  |  |  |  |  |  | 1 |  |
| **c-15t** | P29S |  |  |  |  |  |  | 1 |  |
| **c-15t** | M105I | c-52t |  |  |  |  |  | 1 |  |
| **c-15t** | G169S |  |  |  |  |  |  | 1 |  |
| **c-15t** | M176T |  |  |  |  |  |  | 2 |  |
| **c-15t** | **S315T** |  |  |  |  |  |  | 5 |  |
|  | Q88P+M257V | c-52t |  |  |  |  |  | 1 |  |
|  | W91R |  |  |  |  |  |  | 1 |  |
|  | A122D |  |  |  |  |  |  | 1 |  |
|  | Q127P |  |  |  |  |  |  | 1 |  |
| **g-17t** | L141V | **c-72t** |  |  |  |  |  | 1 |  |
|  | T275P | **g-48a** |  |  |  |  |  | 2 |  |
|  | A312E | c-52t |  |  |  |  |  | 1 |  |
|  | **S315N** |  |  |  |  |  |  | 3 |  |
|  | **S315T** |  |  |  |  |  |  | 55 |  |
| c-34t | **S315T** |  |  |  |  |  |  | 1 |  |
| **t-8c** | **S315T** |  |  |  |  |  |  | 2 |  |
| A190S | **S315T** |  | Q52R |  |  |  |  | 1 |  |
|  | **S315T** |  |  |  | R479P |  |  | 1 |  |
|  | **S315T** |  |  |  | D198G |  |  | 1 |  |
|  | **S315T** |  | A154V |  |  |  |  | 1 |  |
|  | **S315T** |  | M138V |  |  |  |  | 2 |  |
|  | D419Y |  |  |  |  |  |  | 1 |  |
| R27W | P422L | **c-57t** |  |  |  |  |  | 1 |  |
|  | R484H | **g-48a** |  |  |  |  |  | 1 |  |
|  | **Q88*** |  |  |  |  |  |  | 1 |  |
|  | **W198*** |  |  |  |  |  |  | 1 |  |
|  | **713gcg_in+334g_in** |  |  |  |  |  |  | 1 |  |
|  | **2044t_in** |  |  |  |  |  |  | 1 |  |
|  |  | **g-48a** |  |  |  |  |  | 1 |  |
|  |  |  |  |  | K526R |  |  | 1 |  |

The bold mutations are related to phenotypic resistance according to previous studies.

Supplement table 3 The distribution of mutations on *rpoB* in 100 RIF-resistant and 10 susceptible strains.

| *rpoB* mutation (codon position) | No. of isolates with resistant  phenotype=100 | No. of isolates with susceptible phenotype=10 |
| --- | --- | --- |
| **V170F** | 1 |  |
| **V170F+H445Y** | 1 |  |
| **L430P**+**D435G** | 1 |  |
| **L430P**+**D435N** | 1 |  |
| **L430P**+**H445N** | 1 |  |
| **L430P**+S431G | 1 |  |
| **Q432L**+S576L | 1 |  |
| **D435A**+**H445D** | 1 |  |
| **D435A**+**L452P** | 1 |  |
| **D435V** | 13 |  |
| **H445D** | 4 |  |
| **H445L** | 3 |  |
| **H445Q+L452S** | 1 |  |
| **S450F** | 1 |  |
| **S450L** | 62 |  |
| **S450W** | 1 |  |
| **L452P** | 1 |  |
| **S450V+S254P** | 1 |  |
| **S450P** | 1 |  |
| **S450H** | 1 |  |
| H674Y+1291_gcc_in | 1 |  |
| WT | 1 | 10 |

The bold mutations are related to phenotypic resistance according to previous studies.

Supplement table 4 The distribution of mutations on *embB, embA, embC* and *ubiA* in 58 EMB-resistant and 52 susceptible strains.

| embB mutation (codon position) | embA mutation (codon position) | embC mutation (codon position) | embR mutation (codon position) | ubiA mutation (codon position) | No. of isolates with resistant phenotype=58 | No. of isolates with susceptible phenotype=52 |
| --- | --- | --- | --- | --- | --- | --- |
|  | **c-16t** |  |  |  |  | 3 |
|  | **c-8t** |  |  |  |  | 1 |
|  | E951D |  |  |  |  | 2 |
| D328G+**D354A** |  |  |  | M187V | 1 |  |
| **D328Y** |  |  |  |  | 2 |  |
| **D354A** |  |  |  |  | 2 | 1 |
| **D354A**, D1017G |  | V974L |  |  | 1 |  |
| **M306I** |  |  |  |  | 5 | 2 |
| **M306I** |  |  | 54a_del |  |  | 1 |
| **M306I** |  |  |  | M180V | 1 |  |
| **M306I** |  |  |  | R240C | 1 |  |
| **M306I**+A379T+D1024N |  |  |  |  | 1 |  |
| **M306I**+D1024N |  |  |  |  | 2 |  |
| **M306I**+G95S |  |  |  |  | 3 | 1 |
| **M306I**+T667A | **c-12t** |  |  |  | 1 |  |
| **M306I+G406A** |  |  |  |  | 1 |  |
| **M306I+G406D** |  |  |  |  | 1 |  |
| **M306I+Q497R** |  |  |  |  |  | 1 |
| M306L |  |  |  |  | 1 | 1 |
| M306L | **c-16t** |  |  |  | 1 |  |
| M306L+**G406D** |  |  |  |  | 1 |  |
| **M306V** |  |  |  |  | 16 | 3 |
| **M306V** | **c-16t** |  |  |  | 1 |  |
| **M306V** | P2T |  |  |  | 1 |  |
| **M306V**+A201S | E951D |  |  |  | 1 |  |
| **M306V**+G246R |  |  |  |  | 1 |  |
| **M306V**+D1024N |  |  |  |  | 1 |  |
| P404T |  |  | 734c_in | V55L |  | 1 |
| **G406A** |  |  |  |  | 1 | 1 |
| **G406C** | W624R |  |  |  | 1 |  |
| **G406C** | c-16a |  |  |  | 1 |  |
| **G406D** |  |  |  |  | 2 | 1 |
| **G406S** |  |  |  |  |  | 1 |
| **G406S** | E951D |  |  |  | 1 |  |
| I563L |  |  |  |  |  | 4 |
| **Q497K** |  |  |  |  |  | 1 |
| **Q497K** |  |  |  | I179L | 2 |  |
| **Q497P** |  |  |  |  | 1 |  |
| **Q497R** |  |  |  |  | 1 | 1 |
| H1002R | A457E |  |  |  |  | 1 |
|  | G149V |  |  |  |  | 1 |
|  | G275S | g-43c |  |  |  | 1 |
|  | V206M, A542V |  |  |  | 1 |  |
|  | D1049G |  |  |  |  | 1 |
| A679T |  |  |  |  |  | 1 |
|  |  | g-43c |  |  | 1 | 1 |
|  |  | L434S |  |  |  | 1 |
|  |  | V413M |  |  |  | 1 |
|  |  |  | Q221K |  |  | 1 |
|  |  |  |  | F328Y, R282P |  | 1 |
|  |  |  |  | P122R |  | 1 |
| WT | WT | WT | WT | WT |  | 15 |

The bold mutations are related to phenotypic resistance according to previous studies.

Supplement table 5 The distribution of mutations on *pncA, rpsA,* and *panD* in 65 PZA-resistant and 45 susceptible strains.

| *pncA* mutation  (codon position) | *rpsA* mutation  (codon position) | *panD mutation*  *(codon position)* | No. of isolates with resistant phenotype=65 | No. of isolates with susceptible phenotype=45 |
| --- | --- | --- | --- | --- |
| t-11c |  |  | 3 |  |
| L4W |  |  | 4 |  |
| I5T |  |  | 1 |  |
| V9A |  |  | 1 | 2 |
| Q10R |  |  | 1 |  |
| C14W |  |  | 1 |  |
| L19R |  |  | 1 |  |
| L19P |  |  | 1 |  |
| A28D |  |  | 1 |  |
| L35R |  |  |  | 1 |
| Y41* |  |  | 1 |  |
| Y41* | D160A |  | 2 |  |
| A46V |  |  | 1 |  |
| T47A |  |  | 1 |  |
| T47I |  |  |  | 1 |
| T47P |  |  |  | 1 |
| D49G |  |  | 1 |  |
| H51R |  |  | 1 |  |
| P54R |  |  | 1 |  |
| P54R | D266A |  | 1 |  |
| S59Y+T153P |  |  | 1 |  |
| D63G |  |  | 1 |  |
| S67P |  |  | 1 |  |
| C72W |  |  | 2 |  |
| C72Y |  |  | 1 |  |
| T76P |  |  | 2 |  |
| L85R |  |  | 1 |  |
| K96R |  |  | 1 |  |
| K96T |  |  | 2 |  |
| F106C |  |  |  | 1 |
| W119C |  |  | 1 |  |
| V131F | t-66c |  | 1 |  |
| D136G |  |  | 1 |  |
| V139A |  |  | 2 | 1 |
| V139G |  |  | 1 |  |
| V139L |  |  | 2 |  |
| Q141P |  |  | 2 |  |
| T142A |  |  | 1 |  |
| M175R |  |  | 1 |  |
| M175T |  |  | 1 |  |
| T177P | E168K |  | 1 |  |
| 85c_del | A88S |  | 1 |  |
| 134t_del |  |  | 1 |  |
| 281t_in |  |  | 1 |  |
| 399t_in |  |  | 1 |  |
| 420cg_in | T29M |  | 1 |  |
| 528c_del |  |  | 2 |  |
| WT | WT |  | 9 | 38 |

Supplement table 6 The distribution of mutations on *gyrA* and *gyrB* in 65 LFX-resistant and 45 susceptible strains.

| *gyrA* mutation (codon position) | *gyrB* mutation (codon position) | No. of isolates with resistant phenotype=65 | No. of isolates with susceptible phenotype=45 |
| --- | --- | --- | --- |
| G88A+**D94A** |  | 1 |  |
| **A90V** |  | 2 | 9 |
| **A90V** | E501V | 1 |  |
| **A90V** | E501D |  | 1 |
| **A90V** | S477F |  | 1 |
| **A90V** | T500A |  | 1 |
| **A90V** | A504V | 2 |  |
| **A90V+D94A** |  | 1 |  |
| **A90V+D94N** |  | 1 |  |
| **A90V+D94Y** |  |  | 1 |
| **A90V**+D94H |  | 1 |  |
| **A90V+D94G**+I287T |  | 1 |  |
| **S91P** |  | 1 | 2 |
| **S91P+D94A** | V427A | 2 |  |
| **D94A** |  |  | 3 |
| **D94A** | N499Y | 1 |  |
| **D94A**+R309W |  |  | 1 |
| **D94G** |  | 9 | 2 |
| **D94G** | A288D | 1 |  |
| **D94G** | G512R | 1 | 2 |
| **D94G** | D461N |  | 1 |
| D94H |  | 1 | 1 |
| **D94N** |  | 7 |  |
| **D94N** | G512R | 1 |  |
| **D94Y** |  | 1 | 1 |
| G158V | D461N |  | 1 |
| H70R |  |  | 2 |
| I287T |  | 1 |  |
| A288D |  |  | 1 |
| S642C |  |  | 1 |
|  | A504T+M616I |  | 1 |
|  | V636L |  | 1 |
| WT | WT | 2 | 39 |

The bold mutations are related to phenotypic resistance according to previous studies.

Supplement table 7 The distribution of mutations on *gyrA* and *gyrB* in 38 MFX-resistant and 72 susceptible strains.

| *gyrA* mutation (codon position) | No. of isolates with resistant phenotype=38 | No. of isolates with susceptible phenotype=72 |
| --- | --- | --- |
| G88A+**D94A** | 1 |  |
| **A90V** | 5 | 12 |
| **A90V+D94A** | 1 |  |
| **A90V+D94N** | 1 |  |
| **A90V+D94Y** |  | 1 |
| **A90V**+D94H | 1 |  |
| **A90V+D94G**+I287T | 1 |  |
| **S91P** | 1 | 2 |
| **S91P+D94A** | 2 |  |
| **D94A** | 1 | 3 |
| **D94A**+R309W |  | 1 |
| **D94G** | 11 | 5 |
| D94H | 1 | 1 |
| **D94N** | 8 |  |
| **D94Y** | 1 | 1 |
| G158V |  | 1 |
| H70R |  | 2 |
| I287T | 1 |  |
| A288D |  | 1 |
| S642C |  | 1 |
|  |  | 1 |
|  |  | 1 |
| WT | 2 | 39 |

The bold mutations are related to phenotypic resistance according to previous studies.

Supplement table 8 The distribution of mutations on *rrs* and *eis* in 11 KM resistant and 99 susceptible strains.

| *rrs* mutation (nucleotide position) | *eis* mutation (codon position) | No. of isolates with resistant phenotype=11 | No. of isolates with susceptible phenotype=99 |
| --- | --- | --- | --- |
| **a1401g** |  | 8 |  |
| **a1401g**+a514c |  | 2 |  |
|  | **c-10t** |  | 1 |
| a514c |  |  | 2 |
| a1449g |  |  | 1 |
| c1141t |  |  | 1 |
| c1141t+a908c |  |  | 3 |
| c1690t |  |  | 1 |
| g1990a |  |  | 1 |
| g2475t |  |  | 1 |
|  | A86V |  | 2 |
|  | V163I |  | 3 |
|  | S214L |  | 1 |
| WT | WT | 1 | 82 |

The bold mutations are related to phenotypic resistance according to previous studies.

Supplement table 9 The distribution of mutations on *rrs* in 11 AM resistant and 99 susceptible strains.

| *rrs* mutation (nucleotide position) | No. of isolates with resistant phenotype=11 | No. of isolates with susceptible phenotype=99 |
| --- | --- | --- |
| **a1401g** | 8 |  |
| **a1401g**+a514c | 2 |  |
| a514c |  | 2 |
| a1449g |  | 1 |
| c1141t |  | 1 |
| c1141t+a908c |  | 3 |
| c1690t |  | 1 |
| g1990a |  | 1 |
| g2475t |  | 1 |
| WT | 1 | 89 |

The bold mutations are related to phenotypic resistance according to previous studies.

Supplement table 10 The distribution of mutations on *rrs* and *tlyA* in 9 CM resistant and 101 susceptible strains.

| *rrs* mutation  (nucleotide position) | *tlyA* mutation  (codon position) | No. of isolates with resistant phenotype=9 | No. of isolates with susceptible phenotype=101 |
| --- | --- | --- | --- |
| **a1401g** |  | 6 | 2 |
| **a1401g**+a514c |  | 2 |  |
| a514c |  |  | 1 |
| a1449g |  |  | 1 |
| c1141t+a908c |  |  | 3 |
| c1690t |  |  | 1 |
| g1990a |  |  | 1 |
| g2475t |  |  | 1 |
| WT | WT | 1 | 91 |

The bold mutations are related to phenotypic resistance according to previous studies.

Supplement table 11 The distribution of mutations on *rpsL, rrs* and *gidB* in 69 SM-resistant and 41 susceptible strains.

| *rpsL* mutation  (codon position) | *rrs* mutation  (nucleotide position) | *gidB* mutation  (codon position) | No. of isolates with resistant phenotype=69 | No. of isolates with susceptible phenotype=41 |
| --- | --- | --- | --- | --- |
|  |  | **102g_del** | 1 |  |
|  |  | **351g_del** | 1 |  |
|  |  | **386g_in** | 1 |  |
|  |  | A19G |  | 1 |
|  |  | A72T |  | 1 |
|  |  | I81R |  | 1 |
|  |  | P93Q |  | 1 |
|  |  | R97H | 2 |  |
|  |  | H174Y |  | 2 |
| **K43R** |  |  | 45 |  |
| **K43R** |  | **114c_del** | 1 | 1 |
| **K43R** |  | c-66a | 1 |  |
| **K43R** |  | g-15a | 1 |  |
| **K43R** |  | P38R | 1 |  |
| **K43T** | **a514c** |  | 1 |  |
| K88M |  |  | 1 |  |
| **K88R** |  |  | 5 |  |
| K88T |  | **115c_del** | 1 |  |
|  | **a514c** |  | 4 | 1 |
| WT | WT | WT | 3 | 33 |

The bold mutations are related to phenotypic resistance according to previous studies.

Supplement table 12 The distribution of mutations on *inhA* and *ethA* in 23 PTO resistant and 87 susceptible strains.

| *inhA* mutation (codon position) | *ethA* mutation (codon position) | No. of isolates with resistant phenotype=23 | No. of isolates with susceptible phenotype=87 |
| --- | --- | --- | --- |
| t-8c |  |  | 2 |
| **c-15t** | **1114a_del** | 2 |  |
| **c-15t** | G184D | 1 |  |
| **c-15t** | L190P | 1 |  |
| **c-15t** | T342A |  | 1 |
| **c-15t** | W45R | 1 |  |
| **c-15t** | R54H | 1 |  |
| **c-15t** |  | 3 | 4 |
| **c-15t** | t-11c | 1 |  |
| **c-15t** | W116C | 1 |  |
|  | **11a_in** |  | 2 |
|  | **140t_del** |  | 1 |
|  | **675c_in** | 1 |  |
|  | **884t_del** | 1 | 1 |
|  | **1054g_del** | 1 |  |
|  | **1299g_in** |  | 1 |
|  | V17G**+37a_del** |  | 1 |
|  | S40F |  | 1 |
|  | L47S |  | 1 |
|  | F48V |  | 1 |
|  | S55P |  | 1 |
|  | S57P**+Y461*** | 1 |  |
|  | P68L |  | 1 |
|  | S183R**+Y286*** | 1 |  |
|  | T186I | 1 |  |
|  | R227P |  | 1 |
|  | P230R |  | 2 |
|  | Q246P |  | 1 |
|  | S266R |  | 9 |
|  | S266R+a-7c | 1 |  |
|  | **Y286*** |  | 1 |
|  | A341V | 1 | 1 |
|  | **Q359*** |  | 1 |
|  | **W391*** | 1 |  |
|  | S399W | 1 |  |
|  | D464G |  | 1 |
|  | R470C**+11a_in** | 1 |  |
|  | R470C**+808c_in** | 1 |  |
| g-17t | C137R |  | 1 |
| WT | WT |  | 51 |

The bold mutations are related to phenotypic resistance based on a solid association between mutations and resistance reported previously

Supplement table 13 The distribution of mutations on *folC, thyA, ribD, dfrA* in 7 PAS resistant and 103 susceptible strains.

| *folC* mutation (codon position) | *drfA* mutation (codon position) | *thyA* mutation (codon position) | *ribD* mutation (codon position) | No. of isolates with resistant phenotype=7 | No. of isolates with susceptible phenotype=103 |
| --- | --- | --- | --- | --- | --- |
|  | Q28L |  |  |  | 1 |
|  | E90A |  |  |  | 1 |
| F374L |  |  |  |  | 3 |
| G283D |  |  |  |  | 1 |
| **I43T** |  |  |  | 2 |  |
| **I43T** |  | t-31c |  | 1 |  |
|  |  | t-31c |  |  | 1 |
| **S150G** |  |  |  | 2 |  |
| **S150G** |  | **H75N** |  | 1 |  |
| **S150G** |  | A262V |  |  | 1 |
|  |  | S160G |  |  | 1 |
|  |  | 290g_in |  |  | 1 |
| WT | WT | WT | WT | 1 | 93 |

The bold mutations are related to phenotypic resistance based on a solid association between mutations and resistance reported previously

Supplement table 14 The distribution of mutations on *rv0678, rv1979c, rv2535c, mmpL3* and *mmpL5* in 6 CLO resistant and 74 susceptible strains.

| *rv0678* mutation (codon position) | *rv1979c* mutation (codon position) | *rv2535c* mutation (codon position) | *mmpL3* mutation (codon position) | *mmpL5* mutation (codon position) | No. of isolates with resistant phenotype=6 | No. of isolates with susceptible phenotype=74 |
| --- | --- | --- | --- | --- | --- | --- |
| **L32S** |  |  |  |  | 2 |  |
| **R50Q** |  |  |  |  | 1 | 1 |
| **E104G** |  |  |  |  |  | 1 |
| **Q115P** | T265A |  |  |  | 1 |  |
| **V1L** |  |  |  |  |  | 1 |
|  |  |  | W731G |  |  | 1 |
|  |  |  | Q435E |  |  | 1 |
|  |  |  | D152H |  |  | 1 |
|  | R348Q |  |  |  |  | 2 |
|  | V221G |  |  |  |  | 1 |
|  |  |  |  | Q666H |  | 1 |
|  |  |  |  | L367P |  | 1 |
|  |  |  |  | G246S |  | 3 |
|  |  |  |  | H77Y |  | 1 |
|  |  | G306E |  |  |  | 1 |
| WT |  |  |  |  | 2 | 58 |

The bold mutations are related to phenotypic resistance based on a solid association between mutations and resistance reported previously

Supplement table 15 The distribution of mutations on all 34 resistance-related genes in 10 non-MDR strains.

| **Isolates number** | **INH** | **RIF** | **EMB** | **PZA** | **LFX** | **MFX** | **AM** | **KM** | **CM** | **SM** | **PAS** | **PTO** | **CLO** |
| --- | --- | --- | --- | --- | --- | --- | --- | --- | --- | --- | --- | --- | --- |
| 14-1886 |  |  |  |  |  |  |  |  |  |  |  |  |  |
| 14-311 |  |  |  |  |  |  |  |  |  |  |  |  | pepQ c-3t |
| 15-2011 |  |  |  |  |  |  |  |  |  |  |  |  |  |
| 16-2185 |  |  |  |  |  |  |  |  |  |  |  |  |  |
| 16-2291 | inhA c-15t |  |  |  |  |  |  |  |  | GidB A19G |  | inhA c-15t |  |
| 16-5703 |  |  |  |  |  |  |  |  |  |  |  |  |  |
| 16-994 |  |  |  |  |  |  |  |  |  |  |  |  |  |
| 17-6382 |  |  | EmbA_E951D | wt |  |  |  |  |  | RpsL K43R |  |  |  |
| 17-6470 |  |  |  |  |  |  |  |  |  |  |  |  |  |
| B269 |  |  | UbiA_P122R |  |  |  |  |  |  |  |  |  |  |

The grid labeled yellow represents phenotypic resistance.

**References:**

[1] ALLIX-BEGUEC C, ARANDJELOVIC I, BI L, et al. Prediction of Susceptibility to First-Line Tuberculosis Drugs by DNA Sequencing[J]. N Engl J Med, 2018,379(15):1403-1415.

[2] MIOTTO P, TESSEMA B, TAGLIANI E, et al. A standardised method for interpreting the association between mutations and phenotypic drug resistance in Mycobacterium tuberculosis[J]. European Respiratory Journal, 2017,50(6):1701354.

[3] WALKER T M, KOHL T A, OMAR S V, et al. Whole-genome sequencing for prediction of Mycobacterium tuberculosis drug susceptibility and resistance: a retrospective cohort study[J]. Lancet Infect Dis, 2015,15(10):1193-1202.

[4] VILCHEZE C, JACOBS W J. Resistance to Isoniazid and Ethionamide in Mycobacterium tuberculosis: Genes, Mutations, and Causalities[J]. Microbiol Spectr, 2014,2(4):M2-M14.

[5] FARHAT M R, SULTANA R, IARTCHOUK O, et al. Genetic Determinants of Drug Resistance in Mycobacterium tuberculosis and Their Diagnostic Value[J]. American Journal of Respiratory and Critical Care Medicine, 2016,194(5):621-630.

[6] GIRI A, GUPTA S, SAFI H, et al. Polymorphisms in Rv3806c ( ubiA ) and the upstream region of embA in relation to ethambutol resistance in clinical isolates of Mycobacterium tuberculosis from North India[J]. Tuberculosis, 2018,108:41-46.

[7] HE L, WANG X, CUI P, et al. ubiA (Rv3806c) encoding DPPR synthase involved in cell wall synthesis is associated with ethambutol resistance in Mycobacterium tuberculosis[J]. Tuberculosis (Edinb), 2015,95(2):149-154.

[8] LINGARAJU S, RIGOUTS L, GUPTA A, et al. Geographic Differences in the Contribution of ubiA Mutations to High-Level Ethambutol Resistance in Mycobacterium tuberculosis[J]. Antimicrobial Agents and Chemotherapy, 2016,60(7):4101-4105.

[9] TULYAPRAWAT O, CHAIPRASERT A, CHONGTRAKOOL P, et al. Association of ubiA mutations and high-level of ethambutol resistance among Mycobacterium tuberculosis Thai clinical isolates[J]. Tuberculosis (Edinb), 2019,114:42-46.

[10] XU Y, JIA H, HUANG H, et al. Mutations Found in embCAB, embR, and ubiA Genes of Ethambutol-Sensitive and -Resistant Mycobacterium tuberculosis Clinical Isolates from China[J]. Biomed Res Int, 2015,2015:951706.

[11] KHOSRAVI A D, SIROUS M, ABDI M, et al. Characterization of the most common embCAB gene mutations associated with ethambutol resistance in Mycobacterium tuberculosis isolates from Iran[J]. Infect Drug Resist, 2019,12:579-584.

[12] SUN Q, XIAO T, LIU H, et al. Mutations within embCAB Are Associated with Variable Level of Ethambutol Resistance in Mycobacterium tuberculosis Isolates from China[J]. Antimicrobial Agents and Chemotherapy, 2018,62(1).

[13] PANKHURST L J, DEL O E C, VOTINTSEVA A A, et al. Rapid, comprehensive, and affordable mycobacterial diagnosis with whole-genome sequencing: a prospective study[J]. Lancet Respir Med, 2016,4(1):49-58.

[14] ZIGNOL M, CABIBBE A M, DEAN A S, et al. Genetic sequencing for surveillance of drug resistance in tuberculosis in highly endemic countries: a multi-country population-based surveillance study[J]. The Lancet Infectious Diseases, 2018.

[15] YADON A N, MAHARAJ K, ADAMSON J H, et al. A comprehensive characterization of PncA polymorphisms that confer resistance to pyrazinamide[J]. Nat Commun, 2017,8(1):588.

[16] SHI W, CHEN J, FENG J, et al. Aspartate decarboxylase (PanD) as a new target of pyrazinamide in Mycobacterium tuberculosis[J]. Emerg Microbes Infect, 2014,3(8):e58.

[17] ZHANG S, CHEN J, SHI W, et al. Mutations in panD encoding aspartate decarboxylase are associated with pyrazinamide resistance in Mycobacterium tuberculosis[J]. Emerg Microbes Infect, 2013,2(6):e34.

[18] GU Y, YU X, JIANG G, et al. Pyrazinamide resistance among multidrug-resistant tuberculosis clinical isolates in a national referral center of China and its correlations with pncA, rpsA, and panD gene mutations[J]. Diagn Microbiol Infect Dis, 2016,84(3):207-211.

[19] WONG S Y, LEE J S, KWAK H K, et al. Mutations in gidB confer low-level streptomycin resistance in Mycobacterium tuberculosis[J]. Antimicrob Agents Chemother, 2011,55(6):2515-2522.

[20] BROSSIER F, VEZIRIS N, TRUFFOT-PERNOT C, et al. Molecular investigation of resistance to the antituberculous drug ethionamide in multidrug-resistant clinical isolates of Mycobacterium tuberculosis[J]. Antimicrob Agents Chemother, 2011,55(1):355-360.

[21] CHENG V W, LEUNG K S, KWOK J S, et al. Phylogenetic and Structural Significance of Dihydrofolate Synthase (folC) Mutations in Drug-Resistant Mycobacterium tuberculosis[J]. Microb Drug Resist, 2016,22(7):545-551.

[22] ZHANG X, LIU L, ZHANG Y, et al. Genetic determinants involved in p-aminosalicylic acid resistance in clinical isolates from tuberculosis patients in northern China from 2006 to 2012[J]. Antimicrob Agents Chemother, 2015,59(2):1320-1324.

[23] ZHAO F, WANG X D, ERBER L N, et al. Binding pocket alterations in dihydrofolate synthase confer resistance to para-aminosalicylic acid in clinical isolates of Mycobacterium tuberculosis[J]. Antimicrob Agents Chemother, 2014,58(3):1479-1487.

[24] MATHYS V, WINTJENS R, LEFEVRE P, et al. Molecular genetics of para-aminosalicylic acid resistance in clinical isolates and spontaneous mutants of Mycobacterium tuberculosis[J]. Antimicrob Agents Chemother, 2009,53(5):2100-2109.

[25] ISMAIL N, OMAR S V, ISMAIL N A, et al. Collated data of mutation frequencies and associated genetic variants of bedaquiline, clofazimine and linezolid resistance in Mycobacterium tuberculosis[J]. Data Brief, 2018,20:1975-1983.

[26] HARTKOORN R C, UPLEKAR S, COLE S T. Cross-Resistance between Clofazimine and Bedaquiline through Upregulation of MmpL5 in Mycobacterium tuberculosis[J]. Antimicrobial Agents and Chemotherapy, 2014,58(5):2979-2981.

[27] ZHANG S, CHEN J, CUI P, et al. Identification of novel mutations associated with clofazimine resistance in Mycobacterium tuberculosis[J]. J Antimicrob Chemother, 2015,70(9):2507-2510.

[28] ALMEIDA D, IOERGER T, TYAGI S, et al. Mutations in pepQ Confer Low-Level Resistance to Bedaquiline and Clofazimine in Mycobacterium tuberculosis[J]. Antimicrob Agents Chemother, 2016,60(8):4590-4599.

[29] LI W, SANCHEZ-HIDALGO A, JONES V, et al. Synergistic Interactions of MmpL3 Inhibitors with Antitubercular Compounds In Vitro[J]. Antimicrob Agents Chemother, 2017,61(4).
